# A stepped wedge randomised controlled trial assessing the efficacy and patient acceptability of virtual clinical pharmacy in rural and remote Australian hospitals

**DOI:** 10.1186/s12913-024-11740-3

**Published:** 2024-11-11

**Authors:** Shannon Nott, Cristen Fleming, Gerard Hawthorn, Georgina Luscombe, Julaine Allan, Emma Webster, Clare Coleman, Kerrin Palazzi, Joshua Dizon, Alice Munro, Brett Chambers

**Affiliations:** 1 Western NSW Local Health District, Dubbo, NSW Australia; 2 School of Rural Health, Faculty of Medicine and Health, University of Sydney, Dubbo, NSW Australia; 3 Rural Health Research Institute, Charles Sturt University, Orange, NSW Australia; 4 Hunter Medical Research Institute, Newcastle, NSW Australia

**Keywords:** Clinical pharmacy, Healthcare services, Pharmacist, Rural and remote, Telehealth, Telepharmacy, Virtual pharmacy.

## Abstract

**Background:**

Despite medication being the most common healthcare intervention and medication-related incidents being common in hospitals, many rural and remote hospitals in Australia lack onsite pharmacy services due to resource constraints. A Virtual Clinical Pharmacy Service (VCPS) staffed by two senior, rural generalist hospital pharmacists assigned to four hospitals each was implemented in rural and remote facilities to determine whether the VCPS increased adherence to National Safety and Quality Health Service Standards (NSQHS).

**Methods:**

A stepped-wedge randomised controlled trial was employed to sequentially implement a telehealth pharmacy service at one-month intervals in eight hospitals. The primary outcomes were patient-level medication reconciliation completion rates on admission and discharge. Secondary measures evaluated compliance with other NSQHS standards (including Best Possible Medication History, Medication Reconciliation and venous thromboembolism risk assessment), patient outcomes (including representation within 48 h, readmission within 28 days and length of stay), and detection of potential medication-related harms (including pharmacist identified medication related problems, reported medication errors and falls). Patients were invited to complete a patient-reported experience questionnaire. Data were collected from electronic medical records and analysed using mixed logistic regression models to estimate the effectiveness of the VCPS. Antimicrobial usage, falls, and medication errors were analysed at the facility level, while other data were analysed at the patient level.

**Results:**

Compared to control (*n* = 535), patients in the intervention period (*n* = 527) were more likely to have an admission medication reconciliation completed (Odds Ratio (OR) 11.16, 95% confidence interval (CI) 5.59–22.30, *p* < 0.001) in models adjusted for the study period. A similar improvement was observed for discharge medication reconciliation completion (OR 4.07, CI 2.38–6.95, *p* < 0.001), whereas a 33-fold improvement was seen in Best Possible Medication History completion (OR 33.27, CI 17.53–63.14, *p* < 0.001). The VCPS documented 879 medication related problems, with 61% of patients having at least one medication-related problem documented by a pharmacist. There was no change in length of stay, falls, readmission rates or reported medication error rates; however, the study was not powered to detect these changes. Patient feedback was positive and comparable to in-person care, with 95% (179/189) reporting their overall experience as ‘good’ or ‘very good.’ No unintended harms were reported.

**Conclusions:**

The VCPS improved compliance with national standards for medication safety, had high patient acceptability and resulted in the detection of clinically relevant medication-related issues in rural and remote settings. The applicability of virtual pharmacy should be explored in further rural and remote locations in addition to other settings such as metropolitan locations with no onsite clinical pharmacists.

**Ethics number:**

GWHREC 2019/ETH13355.

**Trial registration:**

ANZCTR registration number ACTRN12619001757101. Registered on 11/12/2019. Published trial protocol: *A stepped wedge trial of efficacy and scalability of a virtual clinical pharmacy service (VCPS) in rural and remote NSW health facilities*.

**Supplementary Information:**

The online version contains supplementary material available at 10.1186/s12913-024-11740-3.

## Background

Most people will take at least one medicine to help manage or prevent disease during their lifetime. Medication has undeniably impacted how humanity manages ill health; however, despite their well-intentioned use, medicines are also a leading cause of avoidable harm in healthcare systems globally [1]. Such is the extent of the problem that in 2017, the World Health Organization prioritised “Medication Without Harm” as one of the themes for its Global Patient Safety Initiatives, aiming to reduce medication-related harm by 50% over five years [2].

Methods for addressing medication-related harm, such as inpatient clinical pharmacy services, have been primarily developed by resource-rich and high-activity health services, but have not always been translated to rural environments [3]. Pharmacists are recognised as experts in medication management, and their integration with hospital teams has yielded significant improvements in medication safety globally [4]. Pharmacists are best placed to undertake assessments such as Best Possible Medication History (BPMH) and medication reconciliation, enhance evidence-based medication use and ensure best-practice medication safety processes [5, 6].

Despite this widely acknowledged benefit, many small rural and remote hospitals across Australia lack access to clinical pharmacy services to support patient safety, best-practice medication management and meeting national medication safety standards [3, 7]. Due to geographical challenges and limited patient activity, clinical pharmacy services have not always been translatable to smaller, rural communities [3]. The lack of clinical pharmacy services to address medication-related harm further disadvantages rural and remote Australians, who have higher rates of hospitalisation, mortality and injury compared with those living in metropolitan areas [7–9].

As with other clinical services, virtual care or telehealth can supplement the workforce in rural and remote locations [10–12]. Virtual pharmacy reports in the literature include descriptions of programs and implementation, remote dispensing and review and descriptive publications of professional services and clinical activities [13–16]. Most studies focus on outpatient disease management, with fewer evaluating inpatient or acute care clinical pharmacy services [13, 17, 18]. Additionally, more evidence is needed on the effectiveness and acceptability of virtual pharmacy due to the heterogeneity of study designs and the disease states and outcomes evaluated. This study aims to provide evidence on the effectiveness and acceptability of a Virtual Clinical Pharmacy Service (VCPS) in rural and remote hospitals in New South Wales (NSW), Australia.

## Methods

### Setting

This study was situated in western NSW, Australia, home to an estimated 309,100 people geographically dispersed across remote and remote communities covering almost 450,000 square kilometres. Hospital care in the area is provided through rural referral hospitals (larger hospitals), district hospitals, multipurpose facilities (small hospitals) and nurse-only remote clinics, with onsite pharmacy services available in eight of 47 hospitals in the region. The VCPS was established to address the gap in pharmacy services in the region’s remaining smaller hospitals.

### Design

**Fig. 1:**
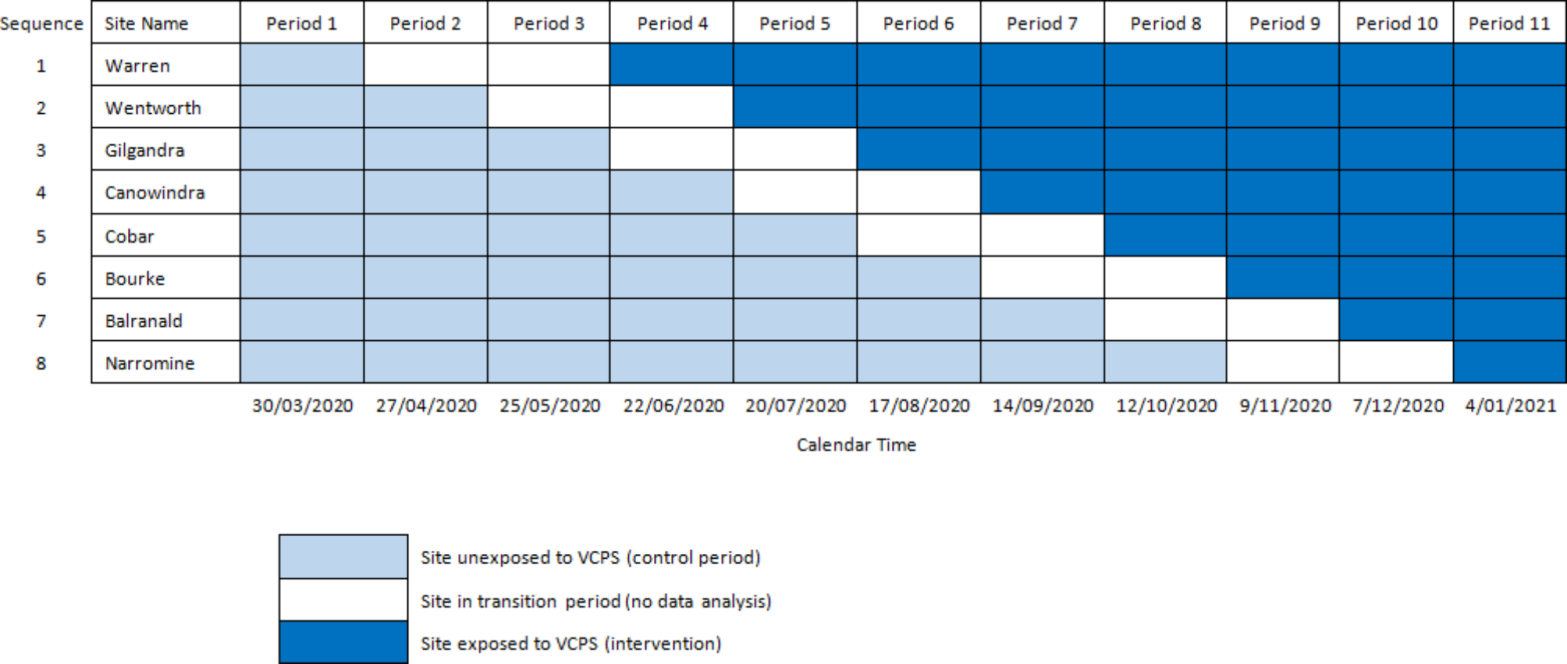
Stepped wedge randomised controlled trial design

This study was a non-blinded mixed-methods study, which employed a stepped-wedge randomised controlled trial design to sequentially implement the VCPS within eight facilities (Fig. 1) [19].

**Fig. 2:**
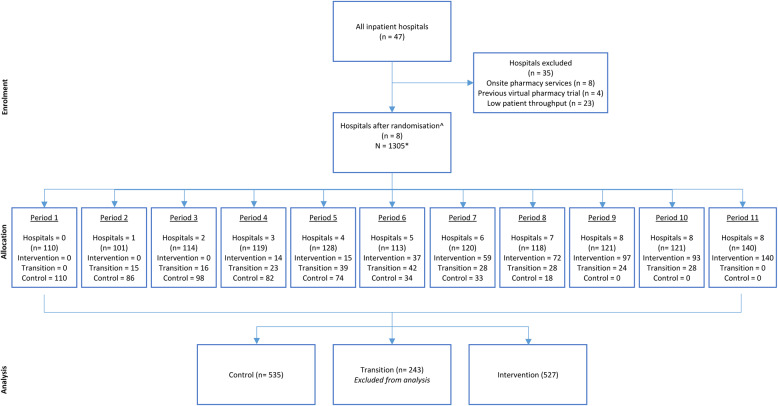
Study flow diagram. Modified for stepped-wedge design from suggested CONSORT criteria format for cluster randomized trials [21]. ^Of the 12 remaining hospitals, 8 were randomised for inclusion in this study. *Patients were deemed eligible if they had a care type of acute and an admission of greater than 24 hours

Rural and remote hospitals within the Far West and Western NSW Local Health District’s without onsite pharmacists or previous participation in a feasibility trial were eligible to participate in the study (Fig. 2). Sites with low patient activity were also excluded to ensure sufficient participants for the intervention. The selected trial hospitals ranged from 12 to 24 inpatient and Emergency Department (ED) beds, 2–7 admitted patients, and 3–10 ED presentations per day in the 2020 and 2021 financial years. One hospital was classified as ‘inner regional’, five as ‘outer regional’, one as ‘remote’ and one as ‘very remote’, using the Accessibility/Remoteness Index of Australia Plus classification system [20]. All patients admitted during the intervention period in ED, inpatient wards and ‘hospital in the home’ wards were able to receive virtual clinical pharmacy services.

Figure 1 details the stepped-wedge randomised controlled trial design, including the “steps” when the trial site moved from the control (pre-VCPS) to the intervention (VCPS). Sites were randomised utilising a computerised random number generator to a step number to control for potential secular confounders. An eight-week transition period was also included in the steps where data were not analysed to allow implementation of the new model of care. The VCPS commenced on 30 March 2020, and data collection was completed on 31 January 2021.

### Intervention

The VCPS provided clinical pharmacy services consistent with recognised standards of practice for hospital pharmacy and undertook activities via videoconferencing, the electronic medical record (eMR) and electronic medication management between 8:00 am and 4:30 pm Monday to Friday [16]. The VCPS consisted of a decentralised team of two senior, rural generalist pharmacists primarily located at the larger regional centres of Dubbo and Orange NSW Australia. Each pharmacist was allocated four hospitals to proactively deliver clinical services virtually but would assist each other during periods of increased service demand. The VCPS activities included BPMH, medication reconciliation at transitions of care, medication review, multidisciplinary team rounds, patient-friendly medication lists (PFML), antimicrobial stewardship and patient and clinician education. Participants were deemed eligible for inclusion for study analysis if they had an admission length of greater than 24 h.

### Process measures

Process measures were collected via the eMR to evaluate service activity, utilisation and uptake of the VCPS (Table 1).

**Table 1:** Process measures to evaluate the utilisation of the Virtual Clinical Pharmacy Service

Number	Process Measure
1	Referred at least once for a pharmacy consult
2	Occasions of care provided
3	Percent of admissions with pharmacist Medication Management Plans
4	Percent of admissions with pharmacist medication reviews
5	Percent of admissions with pharmacist antimicrobial stewardship reviews
6	Percent of admissions with pharmacist discharge medication reviews
7	Percent of admissions with medication education
8	Percent of admissions with smoking histories obtained
9	Percent of smokers prescribed nicotine replacement therapy

### Primary outcome

The primary outcomes (Table 2) were to determine if the VCPS increased admission and discharge medication reconciliation completion. This was quantified by comparing the physician- or pharmacist-completed admission and discharge reconciliation rates in the eMR pre- and post-VCPS intervention.

**Table 2:** Primary and secondary outcome measures

Primary Outcome	Number	Measure
	1	Proportion of admission medication reconciliations completed
	2	Proportion of discharge medication reconciliations completed
*Secondary Outcome*		
Compliance with National Safety and Quality Health Service (NSQHS) Standards for Medication Safety	1	Proportion of admission medication reconciliations completed within KPI (by midnight the following day)
	2	Proportion of patient best-possible medication histories documented during admission
	3	Proportion of patient best-possible medication histories documented within KPI (by midnight the following day)
	4	Proportion of patients who received a patient-friendly medication list (PFML) on discharge
	5	VTE assessment completed by medical officer during admission
Patient medication-related outcomes	6	Proportion of patients with readmission within 28 days of discharge
	7	Count of potential pharmaceutically preventable readmissions
	8	Proportion of patients with an ED presentation not leading to a readmission within 48 h of discharge
	9	Length of Stay
Patient medication knowledge and compliance	10	Medication Adherence Questionnaire [22], change in adherence score between pre (on admission) and post (4 weeks post-discharge)
	11	Patient-reported experience measures survey - responses to the confidence question (question 3)
Patient acceptability	12	Patient-reported experience measures survey - responses to acceptability question (question 1)
	13	Patient-reported experience measures survey - responses to acceptability questions (question 2, question 4, question 5)
Detection of preventable medication harms	14	Rate of falls per 1000 occupied bed days
	15	Count of reported falls incidents
	16	Rate of medication errors per 1000 occupied bed days
	17	Count of medication error incidents reported
	18	Proportion of patients with at least one medication related problem
	19	Rate of pharmacist medication related problems per patient
	20	Proportion of medication related problems by type
	21	The proportion of antimicrobial stewardship review interventions by intervention type
	22	Proportion of VTE recommendations accepted out of all VTE recommendations
	23	Proportion of medication related problems by clinical importance (Minimum - Serious) as assigned by the pharmacist
	24	Uptake of pharmacist recommendations (proportion of actioned medication related problems)

*Abbreviations*: *ED* Emergency Department, *KPI* key performance indicator, *VTE* venous thromboembolism

### Secondary outcome

Secondary outcomes (Table 2) measured the effectiveness and patient acceptability of VCPS. The evaluation used routinely collected data from health information systems pre- and post-VCPS intervention. Secondary outcome measures evaluated compliance with the NSQHS Medication Safety Standard, patient medication-related outcomes, patient medication knowledge and compliance, patient acceptability and detection of preventable medication harms.

### Sample size and analysis

The sample size for the primary outcomes was calculated assuming a referral rate of 29 patients per month, which produced approximately 2088 patients over the stepped-wedge intervention period. A baseline admission and discharge medication reconciliation rate of 11% and an intra-class correlation of 0.05 were assumed, resulting in over 90% power to detect an absolute 10% increase in the proportion of reconciliations performed on admission and discharge, with a type 1 error rate of 2.5%.

Descriptive statistics for categorical data are presented as count (%) or mean (SD) and median (interquartile range; IQR) if continuous. Dichotomous outcome measures were modelled using mixed logistic regression. Fixed effects for VCPS status (Control vs. Intervention) and study period (to account for temporal trends) were included in the model. Random effects for the patient within the facility were included in the modelling to account for correlated observations on the individual level (i.e. patients with repeated admissions) and within-hospital correlations (observations made within a facility). The difference in proportions between Control and Intervention for these outcome measures are reported as odds ratios (OR) with 95% confidence intervals (95% CI). The assumptions for mixed logistic regression were checked for each model and deemed appropriate. The following outcomes were modelled using mixed logistic regression: Primary outcomes 1 and 2 (proportion of completed admission and discharge medication reconciliation), and secondary outcomes 1 (completed medication reconciliation within KPI), 2 (BPMH), 3 (BPMH within KPI), 5 (VTE assessment completion), 6 (readmissions within 28 days), 18 (patients with at least one medication related problem), and 24 (uptake of pharmacist recommendations). Secondary outcomes for VTE assessment completed during admission VTE-related measures (secondary outcome 5) was modelled with an additional adjusting covariate as a fixed effect that accounted for introducing VTE alerts to the health service.

Outcomes measured as discrete counts were modelled using mixed negative binomial regression. Fixed effects for VCPS status and random effects for patients within the facility were included. The difference in counts for these measures is reported as count ratios (CR) with 95% CI. The assumptions for mixed negative binomials were checked for each model and deemed appropriate. Secondary outcomes 9 and 19 were modelled using mixed negative binomial regression.

Continuous outcome measures were modelled using mixed linear regression. Fixed effects for VCPS status and random effects for the facility were included. The difference of these measures between intervention periods is presented as the difference of least square means (LS Mean Difference) with 95% CI. The assumptions for mixed linear regression were checked for each model and deemed appropriate. Secondary outcome 14 was modelled using mixed linear regression.

A subgroup analysis on patients who identified as Indigenous was performed for 28-day readmission and length of stay measures (secondary outcomes 6 and 9). Statistical analyses were programmed using SAS v9.4 (SAS Institute, Cary, North Carolina, USA). A priori, *p* < 0.05 (two-tailed) was used to indicate statistical significance.

Ethical approval was granted by the Greater Western Human Research Ethics Committee prior to the study commencement (2021/ETH00097).

## Results

A total of 1305 eligible acute care patients were admitted during the study period (30 March 2020 to 31 January 2021), with 535 in the control, 243 in transition (transition data collected but not analysed) and 527 in the intervention period. Patient admissions were lower than historical averages during the study, with an average of 118 admissions per period across all sites (range 101–140) (Fig. 2). The median patient age was 74 years, with 53% identified as female and 22% identifying as Aboriginal or Torres Strait Islander. Patient demographics are summarised in Table 3.

**Table 3:** Patient demographics by the stepped-wedge intervention period

Characteristic	Response/Statistic	*Control (n* *= 535)*	*Transition (n* *= 243)*	*Intervention (n* *= 527)*	*Total (N* *= 1305)*
Age (years)	Mean (SD)	68 (18)	68 (18)	70 (18)	68 (18)
	Median (Min, Max)	73 (9, 97)	73 (12, 99)	75 (16, 99)	74 (9, 99)
Sex	Male	235 (44%)	114 (47%)	265 (50%)	614 (47%)
	Female	300 (56%)	129 (53%)	262 (50%)	691 (53%)
Indigenous status	Neither Aboriginal nor Torres Strait Islander	401 (75%)	184 (76%)	427 (81%)	1012 (78%)
	Aboriginal and/or Torres Strait Islander	134 (25%)	59 (24%)	100 (19%)	293 (22%)

During the intervention period, 2193 unique occasions of care were provided by the VCPS, which included medication management plans, medication reviews, antimicrobial stewardship reviews, patient education and smoking history assessment. Among the intervention patients, 28% (145) were referred to the VCPS by onsite hospital staff at least once for pharmacy services. Of all the requests, 94% (440) were marked as complete by the pharmacist. The majority (51%, *n* = 242) of pharmacist consult requests were related to discharge planning. Additional process measures were used to evaluate the utilisation of the VCPS, and the results are presented in Table 4. Data from the control period is included for completeness and represents activity of pharmacists employed at nearby hospitals where their primary role was to supply medications to hospitals participating in the study.

**Table 4:** Process measures of pharmacist clinical activity

Measure	*Control (n* *= 535)*	*Intervention (n* *= 527)*	*Total (N* *= 1062)*
Admissions with pharmacist Medication Management Plans	20 (3.7%)	398 (76%)	418 (39%)
Admissions with pharmacist medication reviews	25 (4.7%)	345 (65%)	370 (35%)
Admissions with pharmacist antimicrobial stewardship reviews	3 (0.6%)	107 (20%)	110 (10%)
Admissions with pharmacist discharge medication reviews	11 (2.1%)	206 (39%)	217 (20%)
Admissions with medication education	10 (1.9%)	196 (37%)	206 (19%)
Admissions with smoking histories obtained	61 (11%)	55 (10%)	116 (11%)

### Primary outcome

The proportion of patients with a medication reconciliation completed by a pharmacist or physician on admission during the intervention period was 85% (450) compared to 44% (234) in the control period. After adjusting for temporal effects, the VCPS intervention resulted in an approximate 11-fold improvement in the odds of a medication reconciliation being completed on admission (*p* < 0.001; Table 5). For discharge reconciliation, there was an approximate four-fold improvement in the odds of a complete discharge medication reconciliation (*p* < 0.001; Table 5). Table 5 also presents findings related to effect on the odds of a `Complete’ medication reconciliation by physician, of which there was a non-significant effect for admission (*p* = 0.910), and a small but significant effect for discharge (*p* = 0.021).

**Table 5:** Primary outcomes for physician and pharmacist completed medication reconciliation on admission or discharge

Primary outcome	Measure	Crude (%)		Adjusted estimate*			Null model ICC
		*Control (n* *= 535)*	*Intervention (n* *= 527)*	OR (95% CI)	*p*-value	*N*	
1 Admission	Medication reconciliation by pharmacist or physician on admission †	234 (44%)	450 (85%)	11.16 (5.59, 22.30)	< 0.001	1062	0.09
	Medication reconciliation by physician on admission	226 (42%)	290 (55%)	1.03 (0.64, 1.64)	0.910	1062	0.07
2 Discharge	Medication reconciliation by pharmacist or physician on discharge †	201 (38%)	356 (68%)	4.07 (2.38, 6.95)	< 0.001	1055	0.14
	Medication reconciliation by physician on discharge	200 (38%)	307 (59%)	1.82 (1.09, 3.02)	0.021	1055	0.16

*Adjusted for the study period

† Composite outcome

### Secondary outcomes

#### Compliance with NSQHS Medication Safety Standards

The VCPS resulted in significant improvements in compliance with NSQHS for Medication Safety. There was a 33-fold increase in the odds of BPMH documentation (*p* < 0.001), and a 20-fold increase in the odds of BPMH completion within 24 h of admission (*p* < 0.001), after accounting for temporal trends (Table 6). VTE assessment completion during admission also showed an increase in odds, but it was not statistically significant (*p* = 0.155). Additionally, the provision of PFML increased from 1.7% during the control period to 34% during the intervention period.

**Table 6:** Compliance with National Safety and Quality Health Service (NSQHS) standards for Medication Safety

Secondary outcome	Measure	Crude (%)		Adjusted estimate*			Null model ICC
		*Control (n* *= 535)*	*Intervention (n* *= 527)*	OR (95% CI)	*p*-value	*N*	
1	Medication reconciliation on admission completed within 24 h †	216 (40%)	292 (55%)	1.24 (0.78, 1.98)	0.356	1062	0.09
2	Best possible medication histories documented during admission	101 (19%)	433 (82%)	33.27 (17.53, 63.14)	< 0.001	1062	0.12
3	Best possible medication histories documented within 24 h	60 (11%)	346 (66%)	19.62 (11.00, 34.99)	< 0.001	1062	0.09
5	VTE assessment completed during admission	111 (21%)	235 (45%)	1.47 (0.86, 2.50) †	0.155	1062	0.10

* Adjusted for study period. † `Partial’ completion of medication reconciliations on admission was counted secondary outcome 1

#### Patient medication-related outcomes

There was no significant impact of the VCPS on overall length of stay (*p* = 0.44) or 28-day readmission (*p* = 0.38). Insufficient data were available to evaluate the effect of the VCPS on ED representations for the same condition within 48 h

#### Patient medication knowledge and compliance

The patient-reported experience measures survey (Table S2) revealed that 91% (164/189) of patients agreed or strongly agreed that they felt more confident managing their medication at home after seeing the pharmacist. However, insufficient data were available to evaluate the effect of the VCPS on change in adherence score using a Medication Adherence Questionnaire.

#### Patient acceptability

The patient-reported experience measures survey (Table S2) demonstrated the VCPS was acceptable to patients. The survey showed that 92% (174/189) of patients reported good or very good audio and video quality, and 96% (182/189) of patients agreed or strongly agreed that pharmacists communicated in a way they could understand. A total of 95% (182/189) of patients reported their overall experience with the pharmacist as good or very good, and 87% (179/189) were likely or highly likely to recommend the service to others. The Patient Reported Experience Measures (PREM) results were comparable to face-to-face pharmacy services at larger referral hospitals.

#### Detection of preventable medication harms

There were insufficient numbers to analyse the effect of the VCPS on staff-reported medication error rates and falls. However, there was an increase in the number of reported medication errors due to increased detection and reporting by pharmacists. No harms or unintended effects due to the VCPS were reported

**Fig. 3:**
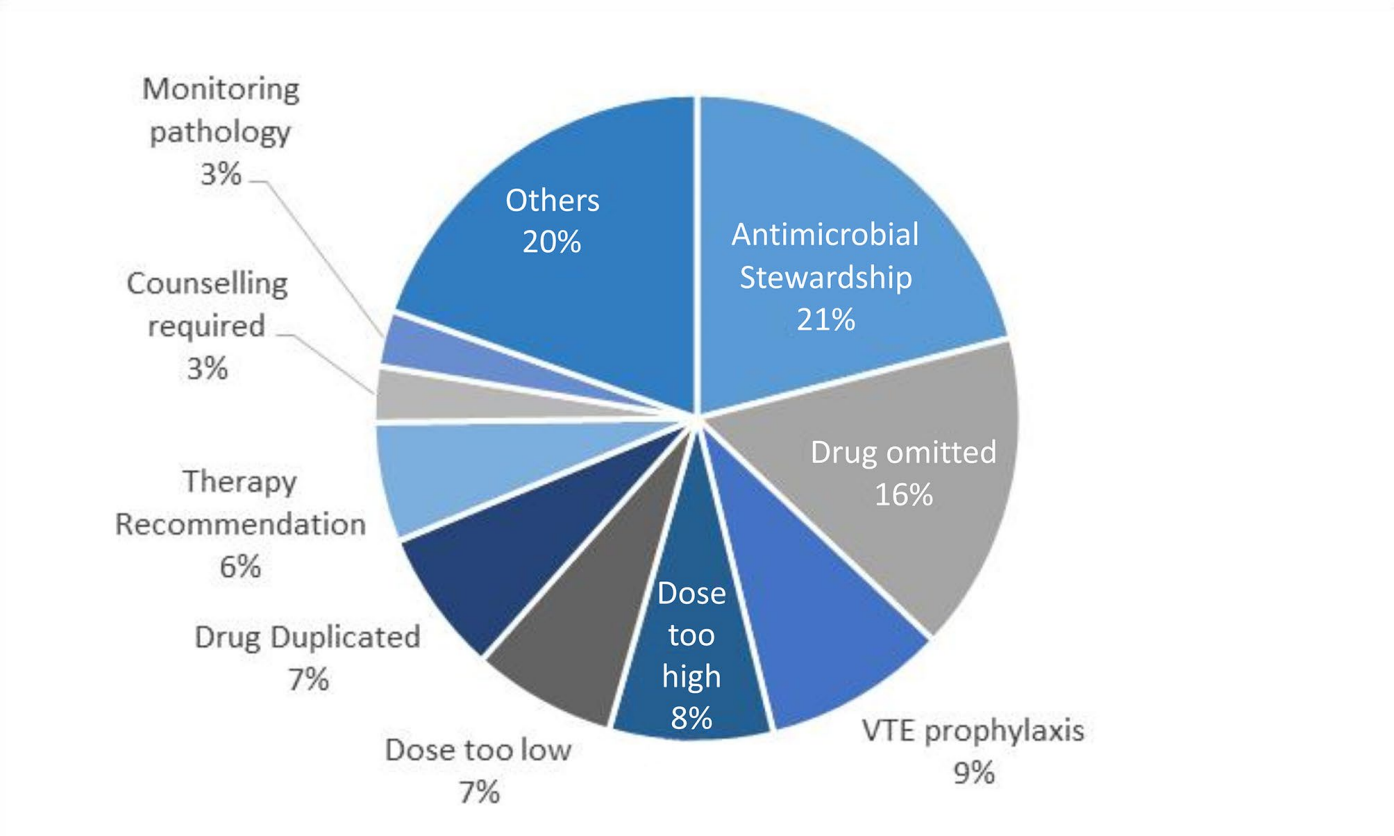
Pharmacist clinical intervention breakdown by type

The VCPS identified 879 medication related problems or recommendations during the intervention period (Fig. 3). Of those, 878 were assigned potential harm rating by the pharmacist with 24% (213) categorised as a minimum, 46% (400) as minor, 29% (258) as moderate, 0.7% (6) as major and 0.1% (1) as serious (Table S1). 61% of patients had at least one medication-related problem documented by a pharmacist, with an average of 1.67 (2.01) per patient. The most common medication related problems were antimicrobial stewardship (21%) and omitted medications (16%), followed by VTE prevention (9.2%) (Fig. 3). Prescribers actioned 73% (587/856) of the pharmacist-identified medication-related problems.

## Discussion

This is the first study in Australia to evaluate the efficacy and patient acceptability of a VCPS in rural and remote hospitals. The authors undertook a robust literature review at initiation of the study which determined there was no similar virtual pharmacy program run in Australia for rural hospitals. This study has broad potential implications, particularly for countries with rural and remote health services that face limited access to clinical pharmacy support. The findings promote the adoption of Virtual Clinical Pharmacy Services (VCPS) as an alternative model for delivering clinical pharmacy care, offering greater efficiency compared to traditional models that depend on extensive travel.

This study contributes to previously reported evidence of high staff acceptability of VCPS in rural and remote [23] and in metropolitan hospitals in Australia [15]. It also adds to the limited body of research evaluating the efficacy and patient acceptability of VCPS in rural and remote hospitals. These findings will be of interest to health service administrators as many rural and remote hospitals struggle to meet NSQHS standards, and the results of this study suggest that VCPS could be a solution for meeting these standards.

### Compliance with NSQHS medication safety standards

The integration of telehealth pharmacists into the healthcare team is a significant step towards improving medication reconciliation and compliance with NSQHS Medication Safety Standards in rural and remote Australian hospitals. The high rate of action by prescribers following pharmacist recommendations points to the value of virtual pharmacists in supplementing the bedside team with accurate and valuable advice for improving patient care.

The study demonstrated meaningful improvements in the odds of medication reconciliation completion by either a pharmacist or medical officer on admission and discharge. Notably, there were significant improvements in BPMH completion and BPMH completion within 24 h of admission, which aligns with the core activities of clinical pharmacists. Although medical officers alone had a significant but smaller increase in discharge medication reconciliation, differing processes for medication reconciliation by pharmacists compared to medical officers in the eMR may have contributed to lower-than-expected reconciliation rates by medical officers. External factors, such as medical workforce shortages and high reliance on ad-hoc, short-term locum medical staff, are likely contributors to these lower reconciliation completion rates; however, this would need to be formally evaluated.

The composite primary outcome combining pharmacist and physician medication reconciliation better reflects the total medication reconciliation activities performed at rural hospitals during the VCPS intervention. The difference in this outcome versus a physician-led reconciliation alone also highlights the opportunity for streamlined medication reconciliation processes within the eMR. Currently, only a physician’s medication reconciliation can be transferred into discharge communication, which means there is still a potential gap in the continuity of medication management and communication of medication changes on transition to the community. Allowing pharmacist reconciliation in the eMR would not only improve compliance with NSQHS standards but also is likely to have significant flow-on benefits to patients by improving communication regarding medicines, reducing errors at transitions of care, and allowing prescribers to make informed treatment decisions based on medication changes while the patient was in hospital [24].

The percentage of patients receiving PFML (37%) was lower than expected but very close to the number of patients reviewed by a pharmacist on discharge (39%). The lack of PFML may be partly due to the nature of virtual pharmacy services. Pharmacists relied on referrals from the on-site healthcare team to capture patients before they went home and there is an absence of reliable indicators in the eMR for pharmacists to self-identify patients who were planned for discharge. The provision of medication lists is a crucial step in providing patient-centred care [25] and is a critical improvement area for the service, with less than half of all patients receiving a PFML. Any improvements will need to consider the timing of patient discharge and the inclusion of VCPS consultation in this complex discharge process [23].

### Detection of medication-related errors

The study revealed several undetected or unreported medication-related errors in small rural hospitals, consistent with findings from studies at larger hospitals with in-person pharmacists [26]. This concordance may suggest that VCPS is as effective as in-person clinical pharmacy. When compared to an earlier Australian study [27], the rate of pharmacist detected medication related problems was 11 times higher in the VCPS intervention, whereas the average rate of detection of 1.67 medication related problems per patient falls within the range of broader literature [3]. Most pharmacist recommendations (70%) were recorded as minimal or minor; however, almost 30% were defined as moderate or above, which could have resulted in a permanent reduction in function, intervention, or increased length of stay if not detected and actioned. A high proportion (73%) of recommendations were actioned by medical officers, suggesting that the recommendations were clinically relevant to patient care.

### Patient acceptability

The delivery of clinical pharmacy through videoconferencing is a significant practice change, especially in the inpatient or ED context, and little was known about its impact on patient experience. Our study has demonstrated that patients found the VCPS to be acceptable, with comparable results to face-to-face services. PREM results suggest that VCPS can provide patient-centred care and establish robust patient-to-provider relationships, even in environments where clinical pharmacists traditionally were not part of the health team. This is likely due to the introduction of dedicated resources for BPMH, medication reconciliation, PFML, and medication education, which involve patients in their care and fill a critical service gap in small rural hospitals.

This is the first study in Australia to evaluate the efficacy and patient acceptability of a VCPS in rural and remote hospitals. This study’s potential impact is wide-reaching, as many countries have rural and remote health services with limited access to clinical pharmacy services, and this research supports the uptake of VCPS as an additional model for clinical pharmacy provision that can provide efficiencies in care provision compared to other traditional models of rural clinical pharmacy reliant on significant travel distances. This study contributes to previously reported evidence of high staff acceptability of VCPS in rural and remote Australian hospitals. It also adds to the limited body of research evaluating the efficacy and patient acceptability of VCPS in rural and remote hospitals. These findings will be of interest to health service administrators as many rural and remote hospitals struggle to meet NSQHS standards, and the results of this study suggest that VCPS could be a solution for meeting these standards.

### Strengths and limitations

This study demonstrated significant improvements in various aspects of clinical pharmacy practice, including BPMH, medication reconciliation, medication review, PFML, and medication error detection. Despite these improvements, no significant changes were observed in patient outcomes such as length of stay and readmissions. The sample size of the study and short timeframe for post-intervention data collection may have contributed to the inability to detect these changes statistically, and it contrasts with other studies that have shown reductions in adverse drug events and hospital revisits with in-person clinical pharmacists [28, 29]. Further research with a larger sample size is needed to determine if the expanded VCPS model can produce similar findings.

A strength of this study was the evaluation of a clinical service in ‘real-world’ conditions over multiple study sites, including hospitals of various sizes. The step-wedge design allowed for a scientifically robust and practical approach, with an adequate timeframe to fully integrate the change into clinical practice. The study team comprised clinicians, clinician-researchers, and research academics who had firsthand experience living and working in rural areas, providing valuable insights into the nuances of the rural environment. Given the findings and “real-world” nature of this study, it is feasible to conclude that this model of care would also benefit other settings where there are no regular clinical pharmacists within acute hospitals.

There are several limitations to take into consideration when interpreting the study results. The limited coding of pharmacist activities and medication-related information in the eMR resulted in a reduced number of measures for evaluation. Improved data collection from the eMR would better capture the full range of pharmacist interventions. Additionally, the variable aptitude for and use of eMR by clinicians may have led to underreporting of activities or medication-related events. The turnover of medical and nursing workforce in some small hospitals further complicated the consistency of documentation and eMR usage. The authors also emphasise that this model relies heavily on real-time documentation within eMR and remote access to patient records, medications, and results. This capability is not widely available globally. While it is feasible to implement the virtual component of this study in non-eMR hospitals (e.g. those using paper records), the authors anticipate that the clinical benefits may be reduced due to the challenges in accessing patient information. A comparative study would be required to assess the extent to which eMR access influences the ability to replicate the outcomes observed in this study.

Quantifying the direct patient benefit of clinical pharmacy interventions is challenging, as baseline medication-related harm data in the study region were limited. Health services historically have variable reporting of baseline medication-related harm, with many studies focusing on chart reviews or undertaking estimates of the total medication related hospital admissions [30, 31]. In the study region, there is limited baseline data to ascertain the level of hospital-related medication harm, making it difficult to determine whether this model impacted this outcome. Further research with larger and longitudinal studies would be necessary to understand the extent of underlying medication-related harm and the potential benefits of the VCPS model. Observation bias may have been introduced through self-reporting of medication related problems by pharmacists; however, the high rate of prescribers acting upon these recommendations suggests their clinical relevance. Additionally, pharmacists self-rated the significant of the interventions that they recorded, which further add potential for bias. Whilst this study did attempt to complete patient PREMS surveys (Table S2), including integrating pre- and post-medication adherence questionnaires [22], the post data have been unable to be interpreted by the research team due to very small numbers (*n* = 8). This is an area identified for future research

The study was also conducted during the COVID-19 pandemic, which placed extraordinary pressure on the healthcare workforce. This pressure and the system-wide changes in healthcare delivery may have positively influenced the engagement of healthcare professionals with the VCPS. The pandemic also brought about changes in health behaviours and increased exposure to virtual care, which could have affected measures of patient acceptability.

## Conclusions

The VCPS has demonstrated its ability to enhance compliance with NSQHS by improving medication-related processes and patient engagement. The acceptability of virtual pharmacy services and the identification of clinically relevant medication-related issues further support its potential in delivering effective and patient-centred care. Virtual pharmacy was acceptable to patients and resulted in the detection of clinically relevant medication-related issues. Future research is required to more adequately determine if virtual pharmacy can improve patient outcomes, such as reducing falls or preventing hospital readmissions.

## Supplementary Information

Supplementary Material 1.

Supplementary Material 2.

Supplementary Material 3.

## Abbreviations

ANZCTR: Australian and New Zealand clinical trials registry
BPMH: Best Possible Medication History
COVID-19: Coronavirus disease 2019
ED: Emergency Department
eMR: Electronic Medical Record
KPI: Key performance indicator
NSQHS: National Safety and Quality Health Service Standards
NSW: New South Wales
PFML: Patient Friendly Medication List
PREM: Patient Reported Experience Measures
VCPS: Virtual Clinical Pharmacy Service
VTE: Venous thromboembolism
