# Supplementary material for: A stepped wedge randomised controlled trial assessing the efficacy and patient acceptability of virtual clinical pharmacy in rural and remote Australian hospitals
Source: BMC Health Serv Res. 2024 Nov 11;24:1375. doi: 10.1186/s12913-024-11740-3 (PMC11552378; doi:10.1186/s12913-024-11740-3)
Supplement: Supplementary file 1 — Supplementary Material 1. [file 12913_2024_11740_MOESM1_ESM.docx]

Table S1: definitions of clinical importance for pharmacist interventions, adapted from Cerner eMR clinical intervention Powerform

| **Clinical Importance** | **When to use** | **Example** | | | | | | |
| --- | --- | --- | --- | --- | --- | --- | --- | --- |
| Serious | *If the intervention did not occur, it may have resulted in the death of the patient* |  |  |  |  |  | Interaction - Be guided by interaction severity rating, i.e. from Mims© | Dose too High, Drug Wrong, Drug Ceased  *Dependent on risk level of medication* |
| Major | *If the intervention did not occur it may have resulted in major permanent loss of function* |  |  |  |  |  |  |  |
| Moderate | *If the intervention did not occur it may have resulted in permanent reduction in function, intervention or increased length of stay* | VTE prophylaxis |  | Omission of a high risk home medication | Drug Contraindicated |  |  |  |
| Minor | *If the intervention did not occur it may have resulted in increased level of care, additional investigations, referral to another clinician* | Therapy recommendation | Medication unavailable |  |  | Omission of a low to moderate risk home medication |  |  |
| Minimum | *The intervention did to have an impact on patient injury, increased level of care or length of stay.* | Counselling required | Allergy update (unless med charted) |  |  |  |  |  |
