# Supplementary material for: A stepped wedge randomised controlled trial assessing the efficacy and patient acceptability of virtual clinical pharmacy in rural and remote Australian hospitals
Source: BMC Health Serv Res. 2024 Nov 11;24:1375. doi: 10.1186/s12913-024-11740-3 (PMC11552378; doi:10.1186/s12913-024-11740-3)
Supplement: Supplementary file 2 — Supplementary Material 2. [file 12913_2024_11740_MOESM2_ESM.docx]

### Table S2:

**Virtual Clinical Pharmacy Service Patient Reported Experience Measures (PREMs) Survey Questions**

| 1 | Did a pharmacist discuss your medicines with you during your stay in hospital? | Yes | No [The selection of No ends the survey] |  |  |  |  |
| --- | --- | --- | --- | --- | --- | --- | --- |
| 2 | If you talked to the pharmacist through videoconferencing, how would you rate the quality of the picture and sound? | very good | good | Neither Good or Poor | poor | very poor | Not Applicable |
| 3 | The pharmacist talked to me in a way I could understand? | Strongly agree | Agree | Neither Agree or Disagree | Disagree | Strongly disagree |  |
| 4 | Following my discussion with the pharmacist, I feel confident in managing my medications at home? | Much more confident | A little more confident | about the same | Less confident | worse than before |  |
| 5 | I was involved as much as I wanted in making decisions about my medications while in hospital? | Strongly agree | Agree | Neither Agree or Disagree | Disagree | Strongly disagree |  |
| 6 | Overall, how would you rate your experience of care with the Pharmacist? | Very good | Good | Neither Good or Poor | Poor | Very Poor |  |
| 7 | How likely are you to recommend the pharmacy service to your friends or family if they were in hospital? | Extremely likely | likely | neither likely or unlikely | unlikely | Extremely unlikely |  |
| 8 | Please select your gender | female | male | Indeterminate / Intersex / Unspecified | Other | prefer not to say |  |
| 9 | What is your age? | 18-54 | 55-64 | 65-74 | 75-84 | 85+ | prefer not to say |
| 10 | Are you of Aboriginal and/or Torres Strait islander origin? | No | Yes, Aboriginal | Yes, Torres Strait Islander | Yes, Aboriginal and Torres Strait Islander |  |  |
| 11 | Would you like to make a… | Compliment | Suggestion | Complaint | Nothing to add |  |  |
| 12 | Please enter your feedback in the space provided | [Free text] |  |  |  |  |  |
